# Astragaloside IV Attenuates Acetaminophen-Induced Liver Injuries in Mice by Activating the Nrf2 Signaling Pathway

**DOI:** 10.3390/molecules23082032

**Published:** 2018-08-14

**Authors:** Lei Li, Wenxiang Huang, Shoukai Wang, Kecheng Sun, Wenxue Zhang, Yanmei Ding, Le Zhang, Bayaer Tumen, Lili Ji, Chang Liu

**Affiliations:** 1 The Shanghai Key Laboratory of Compound Chinese Medicines and MOE Key Laboratory for Standardization of Chinese Medicines, Institute of Chinese Materia Medica, Shanghai University of Traditional Chinese Medicine, Shanghai 201203, China; lil@ahstu.edu.cn; 2 Key Laboratory of Quality & Safety Control for Pork, Ministry of Agriculture and Rural, College of Animal Science, Anhui Science and Technology University, Fengyang 233100, China; huangwx8@zju.edu.cn (W.H.); 18855033867@163.com (S.W.); SL586364@163.com (K.S.); 18855034644@163.com (W.Z.); 18855037810@163.com (Y.D.); zl18855033859@163.com (L.Z.); 3 Key Laboratory of Animal Virology of Ministry of Agriculture and Rural, College of Animal Science, Zhejiang University, Hangzhou 310058, China; 4 Veterinary Laboratory, Shanxi Animal Disease Control Center, Taiyuan 030027, China; Tumenbayaer@163.com

**Keywords:** Astragaloside IV, anti-oxidation, acetaminophen, liver injury

## Abstract

Acetaminophen (APAP) is a well-known antipyretic and analgesic drug. However, the accidental or intentional APAP overdose will induce liver injury and even acute liver failure. Astragaloside IV (AS-IV), a bioactive compound isolated from Astragali Radix, has been reported to have protective effects on the digestive and immune systems because of its anti-oxidant and anti-inflammatory properties. This study aims to observe whether AS-IV pretreatment provides protection against APAP-induced liver failure. The results of serum alanine/aspartate aminotransferases (ALT/AST) analysis, hepatic glutathione (GSH), and malondialdehyde (MDA) amounts, and liver superoxide dismutase (SOD) activity showed that AS-IV protected against APAP-induced hepatotoxicity. Liver histological observation further evidenced this protection provided by AS-IV. AS-IV was found to reverse the APAP-induced increased amounts of pro-inflammatory cytokines, including interleukin 1β (IL-1β), interleukin 6 (IL-6) and tumor necrosis factor alpha (TNF-α). Western-blot analysis showed that AS-IV increased the transcriptional activation of nuclear factor erythroid 2-related factor 2 (Nrf2), and enhanced the expression of heme oxygenase 1 (HO-1) and reduced nicotinamide adenine dinucleotide phosphate (NAD(P)H): quinone oxidoreductase 1 (NQO1) in the presence of APAP. AS-IV also decreased the expression of kelch-like ECH-associated protein-1 (Keap1). In conclusion, we demonstrated that AS-IV exerted a strong protection against APAP-induced hepatotoxicity by activating Nrf2 antioxidant signaling pathways.

## 1. Introduction

Liver injuries triggered by chemical medicines, known as drug-induced liver injury (DILI), is a kind of familiar and severe adverse drug reactions contributing to acute liver failure (ALF). DILI has proved to be the main reason for ALF in America and the United Kingdom, including some other countries and regions [1]. On the basis of statistics gained from the World Health Organization (WHO), DILI has turned out to be the fifth fatal cause of death on a worldwide scale [2].

Acetaminophen (APAP), an extensively applied antipyretic and analgesic drug clinically worldwide, can lead to severe acute liver injury when an unexpected or intentional overdose was taken [3]. Within the range of therapeutic dose, a majority of APAP catalyzed by the cytochrome P450 metabolic enzymes yield the chemically reactive intermediate product, *N*-acetyl-*p*-benzoquinoneimine (NAPQI), which can be rapidly detoxified by combining with glutathione (GSH) under normal circumstances. Nevertheless, once APAP is beyond the normal dosage, an excessive NAPQI deplete hepatic GSH content appears and interacts with critical intracellular proteins, contributing to elevated oxidative stress, severe inflammatory response, and the subsequent hepatocyte necrosis [4,5]. Therefore, developing a notably efficient treatment for acute liver injury is increasingly more and more important for patients [6].

**Figure 1:**
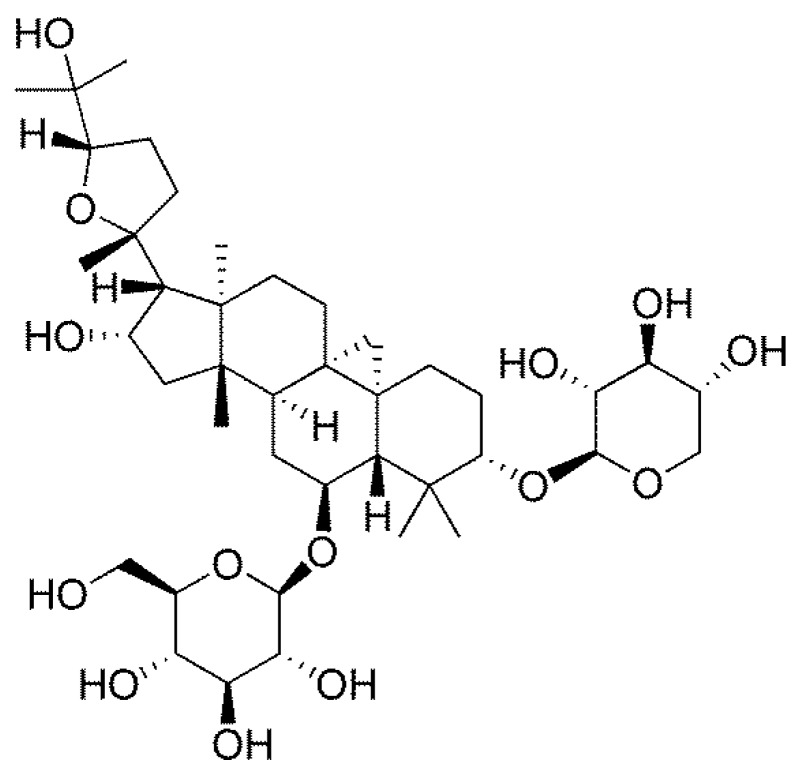
The chemical structure of AS-IV.

Astragaloside IV (AS-IV, Figure 1), a natural bioactive compound isolated from *Astragalus membranaceus Bunge*, is regarded as the critical marker to evaluate the quality of *Astragalus membranaceus* in the Chinese Pharmacopeia, and it has been reported to have protective effects on cardiovascular, digestive, and immune systems because of its evident anti-oxidant and anti-inflammatory properties [7,8]. It has been widely acknowledged that the appearance of oxidative stress and subsequently inflammatory response plays critical roles in the hepatotoxicity induced by APAP [9]. However, the amelioration of AS-IV on APAP-induced liver injury remains unclear. In the present study, we observed the protective effects of AS-IV against APAP-induced hepatotoxicity, and further explore the engaged mechanisms from inhibiting inflammatory and oxidative damage.

## 2. Results

### 2.1. AS-IV Attenuates APAP-Induced Elevation of Serum Alanine/Aspartate Aminotransferase (ALT/AST)

**Figure 2:**
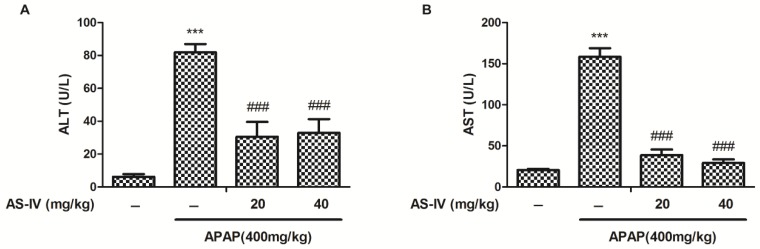
The AS-IV decreased APAP-induced increase in serum ALT (**A**) and AST (**B**) activities. Data were expressed as means ± standard error (SEM) (*n* = 10). *** *p* < 0.001 compared to the vehicle control; ^###^ *p* < 0.001 compared to APAP.

To investigate the hepatoprotective potential of AS-IV, ICR mice were treated with AS-IV (20 and 40 mg/kg) for 7 days before oral administration of 400 mg/kg of APAP. As shown in Figure 2, biochemical indicators of hepatocytes damage, serum ALT and AST, significantly increased 4 h after APAP administration (*p* < 0.001). Whereas pretreatment with AS-IV extremely eliminated APAP-induced hepatotoxicity by decreasing ALT and AST (*p* < 0.001).

### 2.2. Histopathology of the Liver

**Figure 3:**
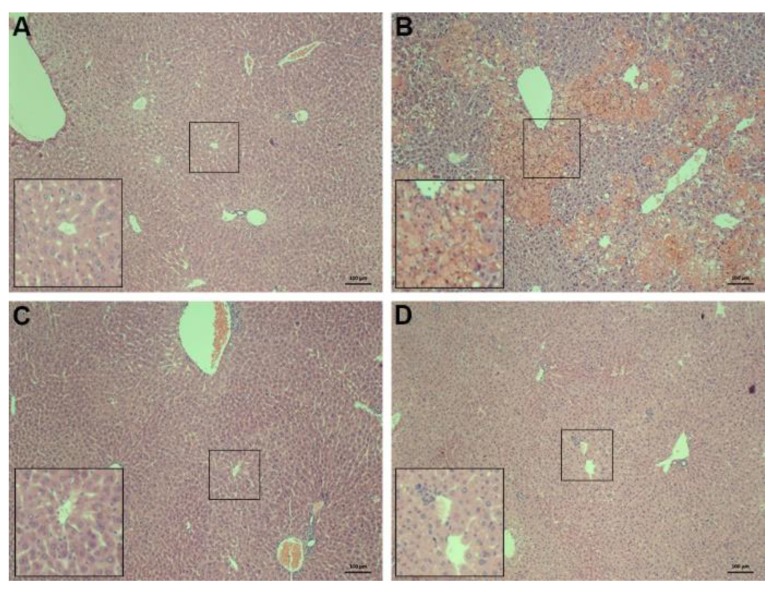
The histological observation of liver injury. After given APAP for 4 h, the livers were removed, fixed, sectioned (5 μm), and processed for hematoxylin and eosin (H&E) staining. Typical images were chosen from each experimental group (*n* = 6). (**A**) Vehicle control, (**B**) APAP (400 mg/kg), (**C**) APAP (400 mg/kg) + AS-IV (20 mg/kg), (**D**) APAP (400 mg/kg) + AS-IV (40 mg/kg) (magnification × 100).

To further evaluate the hepatic protection of AS-IV, a liver histopathology was observed. Mice treated with APAP (400 mg/kg) showed characteristic histologic feature of changes in liver necrosis and inflammation (Figure 3B). AS-IV pretreatment apparently alleviated APAP-induced liver damage (Figure 3C,D).

### 2.3. AS-IV Suppressed APAP-Induced Oxidative Stress

**Figure 4:**
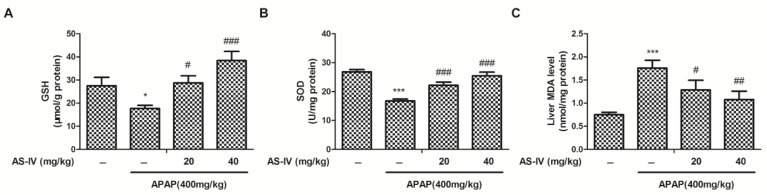
The AS-IV inhibited APAP-induced oxidative stress in liver. After given APAP for 4 h, liver samples were harvested for the GSH amount (**A**), SOD activity (**B**) and MDA content (**C**) evaluation. Data were expressed as means ± SEM (*n* = 10). * *p* < 0.05, *** *p* < 0.001 compared to vehicle control; ^#^ *p* < 0.05, ^##^ *p* < 0.01, ^###^ *p* < 0.001 compared to APAP.

Excessive NAPQI deplete hepatic GSH leads to oxidative injury, which is an irreversible step for APAP-induced hepatocellular necrosis [10]. To explore the antioxidation of AS-IV in APAP-induced liver injury, the hepatic oxidative stress indexes, such as GSH, superoxide dismutase (SOD), and malondialdehyde (MDA), were measured. Liver GSH amount and SOD activity were evidently decreased after APAP (400 mg/kg) intoxication (Figure 4A,B), whereas AS-IV reversed such a decrease (*p* < 0.01, *p* < 0.001). Furthermore, the result of liver MDA assay indicated that APAP (400 mg/kg) exposure significantly increased the amount of MDA, and the increase in MDA was suppressed by the administration of AS-IV (Figure 4C). The above results suggested that APAP-induced oxidative stress could be repressed by AS-IV.

### 2.4. AS-IV Suppressed APAP-Induced Inflammatory Injury

**Figure 5:**
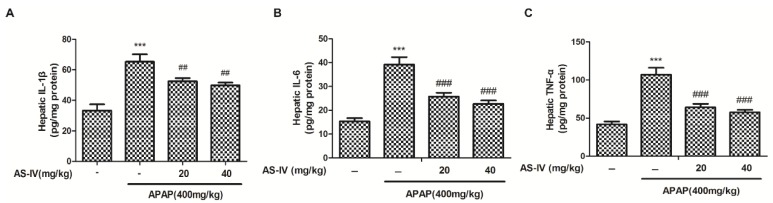
AS-IV repressed APAP-induced increase in hepatic inflammatory cytokines. After given APAP for 4 h, liver samples were harvested for IL-1β (**A**), IL-6 (**B**) and TNF-α (**C**) ELISA assay. Data were expressed as means ± SEM (*n* = 10). *** *p* < 0.001 compared to vehicle control; ^##^ *p* < 0.01, ^###^ *p* < 0.001 compared to APAP.

Inflammatory response plays crucial roles in APAP-induced liver injury [11], thus, the hepatic tumor necrosis factor α (TNF-α), interleukin 1β (IL-1β) and interleukin 6 (IL-6) levels were measured by enzyme linked immunosorbent assays (ELISA) kits. As shown in Figure 5, TNF-α, IL-1β and IL-6 levels were significantly increased in the livers of APAP-treated mice (*p* < 0.001). Whereas, AS-IV potently suppressed the TNF-α, IL-1β, and IL-6 levels induced by APAP (*p* < 0.01, *p* < 0.001). The data suggested that AS-IV inhibited APAP-induced hepatic inflammation.

### 2.5. AS-IV StimulateNrf2 Pathway to Protect APAP-Induced Liver Injury

**Figure 6:**
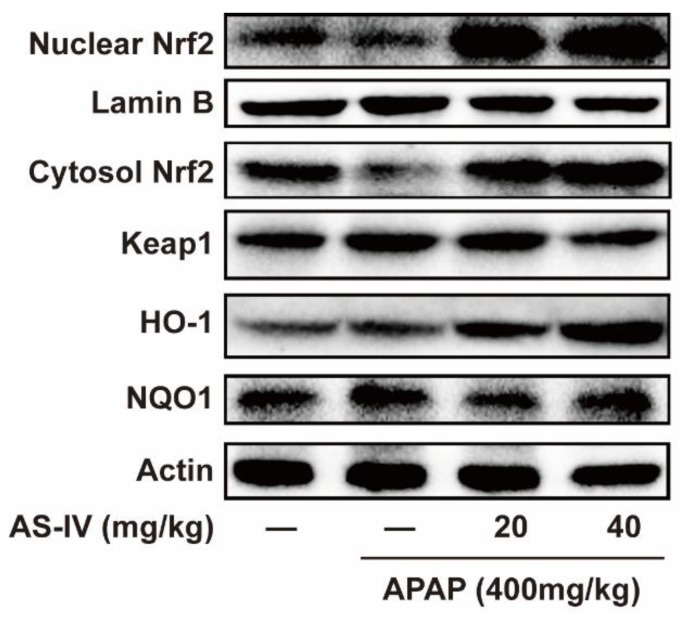
AS-IV activated Nrf2 signaling pathway (*n* = 3). Western blotting analysis of Nrf2, Keap1, HO-1, and NQO1.

In order to determine the possible mechanisms for the onset of the antioxidant, western blotting was used to examine the effects of AS-IV on the Nrf2 signaling pathway. The results of Nrf2 nuclear translocation shown in Figure 6 indicated that nuclear Nrf2 expression was obviously enhanced in mice treated with AS-IV (20 and 40 mg/kg). Next, we observed the Nrf2-regulated downstream gene HO-1 and NQO1, known as anti-oxidative enzymes [12]. As shown in Figure 6, the protein expression of HO-1 and NQO1 increased in mice treated with AS-IV (20 and 40 mg/kg) compared with mice treated with only APAP. These results demonstrated that Nrf2 regulated HO-1 and NQO1 joined in the protection of AS-IV against hepatotoxicity. Keap1 is involved in the process of ubiquitination and degradation of Nrf2, and plays a negative regulation of Nrf2 activity, therefore, the effects of AS-IV on Keap1 expression was detected. Data in Figure 6 showed that AS-IV triggered a remarkably decreased expression of Keap1, which may contribute to the activation of Nrf2. 

## 3. Discussion

AS-IV is one of the major bioactive substances of Astragali Radix, which is usually used for improving immunity and treating diabetes in China. In addition to its immunoregulation, increasing evidence supports AS-IV in the treatment of organ fibrosis, inflammatory injury, oxidative damage, and apoptosis [13]. In this study, we found that AS-IV pretreatment (20 and 40 mg/kg) remarkably ameliorated APAP-induced liver injury, as evidenced by the reduced elevation of ALT and AST (Figure 2), and the alleviated histological abnormalities (Figure 3). There are an increasing number of studies showing that herbal medicines derived from plant extracts are being increasingly utilized to treat liver injury [14,15]. Plant extracts contribute to a variety of intracellular signaling cascades in hepatocyte to ameliorate inflammatory reactions. Moreover, many compounds from natural products, such as chlorogenic acid, andrographolide, and quercetin, have been shown to prevent DILI through their anti-oxidative properties [16,17,18]. Likewise, AS-IV also exhibits the attenuation of oxidative stress. However, few studies have shown the role of AS-IV in APAP-induced liver injury. Our data indicated that AS-IV significantly increased the levels of GSH and SOD and decreased the level of MDA induced by APAP (Figure 4), which suggested that AS-IV may inhibit APAP-induced liver injury via attenuating liver oxidative damage.

Numerous studies reported that the excessive NAPQI, the reactive metabolite of APAP in hepatocytes, depletes cellular GSH promoting uncontrolled reactive oxygen species (ROS) generation, which could induce oxidative injury [19]. The state of oxidative stress can additively increase if the endogenous antioxidant system is not capable of coping with the continuous ROS production [20]. Therefore, the signaling pathways involved in anti-oxidative stress are investigated as attractive targets in the detoxification of APAP. As the most important anti-oxidative transcription factor, Nrf2 plays a critical role in protecting against APAP-induced hepatotoxicity [21]. Our results showed that AS-IV pretreatment enhanced the expression of Nrf2 in the nucleus (Figure 6), which is a symbol of the activation of Nrf2. HO-1 and NQO1, as anti-oxidative enzymes, are regulated when Nrf2 binds to antioxidant-related elements [12,22]. It appears that HO-1 and NQO1 were up-regulated by AS-IV compared to APAP-induced hepatic injury (Figure 6). Keap1 is known as an inhibitor of Nrf2, which contributes to the degradation of Nrf2 by ubiquitination, thus, Keap1 degradation could promote Nrf2 activation by enhancing its nuclear translocation and DNA binding activity [23]. Our data showed that AS-IV reversed the APAP-induced increase of protein expression of Keap1, and this may contribute to the AS-IV mediated-activation of Nrf2. 

It is known that a sterile inflammatory response also plays crucial roles in the progression of APAP-induced hepatotoxicity [24]. Thus, excessive pro-inflammatory cytokines, such as IL-1β, IL-6, TNF-α, were considered as precursors of APAP-induced liver injury, and the down-regulating these pro-inflammatory cytokines were considered as an effective strategy for therapy in APAP-induced hepatic damage. As the most important cytokines in APAP-induced liver injury, the expression of IL-1β, IL-6, and TNF-α were regulated by nuclear factor κB (NF-κB) [25]. After being triggered by ROS, NF-κB translocated from the cytoplasm to the nucleus, thus, activating the expression of pro-inflammatory cytokines involved in the inflammatory process [26]. AS-IV has been demonstrated to exert anti-inflammatory property by suppressing the production of IL-1β, IL-6, and TNF-α in various inflammatory models [27,28]. In the present study, we found that AS-IV reversed the increased pro-inflammatory cytokines IL-1β, IL-6, and TNF-α in liver tissue (Figure 5). Taken together, AS-IV exhibited anti-inflammatory activity in APAP-induced liver injury.

## 4. Materials and Methods

### 4.1. Chemical Compounds and Reagents

AS-IV and APAP were obtained from Shanghai Aladdin Biochemical Technology Co., Ltd. (Shanghai, China), and the purity of the two kinds of reagents is >98.0%. Kits used for detecting ALT, AST, GSH, MDA and SOD were obtained from Nanjing Jiancheng Bioengineering Institute (Nanjing, China). NE-PER nuclear and cytoplasmic extraction reagents, T-PER tissue extraction reagent, SuperSignal West Pico PLUS Substrate kit and Pierce BCA Protein Assay Kit were obtained from ThermoFisher Scientific (Waltham, MA, USA). ELISA kits for murine TNF-α, IL-1β, and IL-6 were obtained from Neobioscience (Shenzhen, China). The antibodies used for immunoblotting anti-Keap1 was purchased from Cell Signaling Technology (Danvers, MA, USA) (1:1000 dilutions). Antibodies used for immunoblotting including anti-Nrf2, -HO-1, and -NQO1 were all bought from Santa Cruz (Santa Cruz, CA, USA) (all 1:200 dilutions). Antibodies used for immunoblotting including anti-Actin and -Lamin B were purchased from Proteintech Group (Wuhan, China) (1:2000 dilutions for Actin, 1:5000 dilutions for Lamin B). Peroxidase-conjugated goat anti-rabbit immunoglobulin IgG (H + L), anti-mouse IgG (H + L) and anti-goat IgG (H + L) were obtained from the Proteintech Group (Wuhan, China). Other reagents, unless indicated, were obtained from Sigma Chemical Co. (St. Louis, MO, USA).

### 4.2. Animals and Treatments

Specific pathogen-free male ICR mice (20 ± 2 g body weight) were obtained from Qinglongshan Laboratory Animal Center (Nanjing, China). The animals were fed with a standard laboratory diet and ample water at room temperature with a 12 h light-dark cycle with 60 ± 10% humidity. All animals received humane care in line with the institutional animal care guidelines approved by the Experimental Animal Ethical Committee (AK-DK-2017026, Approval date: April 25, 2017), Anhui Science and Technology University.

Forty mice were randomly separated into 4 groups each containing 10 mice: (1) Vehicle control (0.5% carboxymethylcellulose sodium), (2) APAP (400 mg/kg), (3) APAP (400 mg/kg) + AS-IV (20 mg/kg), and (4) APAP (400 mg/kg) + AS-IV (40 mg/kg). Mice were pre-administered orally with two different concentrations of AS-IV (20 and 40 mg/kg per day) for 7 consecutive days. To build the liver injury model, mice were orally given a toxic dose of APAP (400 mg/kg) after administration of AS-IV for 1 h on the 7th day [29]. Animals were then sacrificed 4 h after APAP intoxication, then the samples of plasma and liver tissue were collected and stored at −80 °C for further analysis.

### 4.3. Biochemical Analysis of Serum ALT and AST Activities

The blood samples obtained were quietly kept at room temperature for 2 h. Then serum was collected by centrifugation at 1500× *g* for 10 min. Serum ALT and AST were measured with kits according to the manufacturer’s instructions.

### 4.4. Liver Histological Examination

Tissue slices harvested from the same place of the liver in mice were fixed in 10% phosphate-buffered saline-formalin for at least 24 h and then embedded in paraffin for a histological check of the liver tissue damage. Samples were subsequently sectioned (5 μm), stained with H&E, and then observed under a light microscope (Motic VM, Xiamen, China) to appraise liver injury.

### 4.5. Analysis of Liver GSH and MDA Amount

The contents of the liver GSH and MDA were exactly determined according to the manufacturer’s instruction.

### 4.6. Analysis of Liver SOD Activity

Liver SOD enzymatic activity was exactly determined according to the manufacturer’s instructions. Protein concentration was detected by BCA kit and SOD activity was expressed as μmol/g protein.

### 4.7. Analysis of Serum and Hepatic TNF-α, IL-1β and IL-6 Levels

The liver tissues were collected 4 h after APAP treatment. Then the liver samples were weighed and homogenized with saline (1:9, *w*/*v*). The levels of hepatic inflammatory cytokines such as TNF-α, IL-1β, and IL-6 were measured using commercial ELISA kits referring to the manufacturer’s protocols.

### 4.8. Protein Extraction

Liver tissue cellular proteins were isolated using a T-PER tissue extraction reagent or NE-PER nuclear and cytoplasmic extraction kit referring to the manufacturer’s protocols. Protein concentration was detected by BCA kit, and all the samples in the same experiment were normalized to the same protein concentration.

### 4.9. Western-Blot Analysis

Protein samples extracted from liver tissue were separated by 10% sodium dodecyl sulfate–polyacrylamide gel electrophoresis for 2.5 h and electrophoretically transferred to polyvinylidene difluoride membrane for 90 min, then the membranes were blocked with 5% non-fat milk or 5% BSA for 1 h at room temperature. Next, the membranes were incubated with primary antibodies at 4 °C overnight. Posterior to washing the membranes three times with TBST, the membranes were probed with horseradish peroxidase-conjugated secondary antibodies, then the proteins in the membranes were visualized by SuperSignal West Pico PLUS Substrate kit (Waltham, MA, USA) after washing the membranes three times with tris-buffered saline with Tween again. 

### 4.10. Statistical Analysis

Data were expressed as means ± SEM. Multiple comparisons among different groups were conducted by one-way analysis of variance with LSD post-test, and all data were analyzed by SPSS 23.0 (IBM, New York, NY, USA), *p* < 0.05 was defined as statistically significant differences.

## 5. Conclusions

We explored the protective effects of AS-IV in APAP-induced liver injury. Our results showed that the hepatoprotective effect of AS-IV was associated with its strong anti-oxidative and anti-inflammatory properties by activating the Nrf2 signaling pathway. These data strongly indicated that AS-IV may be a promising therapeutic candidate for the treatment of DILI.

## Abbreviations

The following abbreviations are used in this manuscript:

**ALF**	Acute liver failure	**Keap1**	Kelch-like ECH-associated protein-1
**ALT/AST**	Alanine/aspartate aminotransferase	**MDA**	Malondialdehyde
**APAP**	Acetaminophen	**NAPQI**	*N*-acetyl-*p*-benzoquinoneimine
**AS-IV**	Astragaloside IV	**NF-κB**	Nuclear factor kB
**DILI**	Drug-induced liver injury	**NQO1**	NAD(P)H: quinone oxidoreductase 1
**GSH**	Glutathione	**Nrf2**	Nuclear factor erythroid 2-related factor 2
**HO-1**	Heme oxygenase 1	**SOD**	Superoxide dismutase
**IL-1β, 6**	Interleukin 1β, 6	**TNF-α**	Tumor necrosis factor α
